# Hypophosphatemia promotes lower rates of muscle ATP synthesis

**DOI:** 10.1096/fj.201600473R

**Published:** 2016-06-23

**Authors:** Dominik H. Pesta, Dimitrios N. Tsirigotis, Douglas E. Befroy, Daniel Caballero, Michael J. Jurczak, Yasmeen Rahimi, Gary W. Cline, Sylvie Dufour, Andreas L. Birkenfeld, Douglas L. Rothman, Thomas O. Carpenter, Karl Insogna, Kitt Falk Petersen, Clemens Bergwitz, Gerald I. Shulman

**Affiliations:** * Department of Internal Medicine, Yale University School of Medicine, New Haven, Connecticut, USA;; † Department of Cellular and Molecular Physiology, Yale University School of Medicine, New Haven, Connecticut, USA;; ‡ Department of Radiology and Biomedical Engineering, Yale University School of Medicine, New Haven, Connecticut, USA;; § Howard Hughes Medical Institute, Yale University School of Medicine, New Haven, Connecticut, USA;; ¶ Department of Pediatrics, Yale University School of Medicine, New Haven, Connecticut, USA; and; ‖ Novo Nordisk Foundation Center for Basic Metabolic Research, University of Copenhagen, Copenhagen, Denmark

**Keywords:** [^31^P]MRS, saturation transfer, inorganic phosphate

## Abstract

Hypophosphatemia can lead to muscle weakness and respiratory and heart failure, but the mechanism is unknown. To address this question, we noninvasively assessed rates of muscle ATP synthesis in hypophosphatemic mice by using *in vivo* saturation transfer [^31^P]-magnetic resonance spectroscopy. By using this approach, we found that basal and insulin-stimulated rates of muscle ATP synthetic flux (*V*_ATP_) and plasma inorganic phosphate (P_i_) were reduced by 50% in mice with diet-induced hypophosphatemia as well as in sodium-dependent P_i_ transporter solute carrier family 34, member 1 (*NaPi2a*)-knockout (*NaPi2a*^−/−^) mice compared with their wild-type littermate controls. Rates of *V*_ATP_ normalized in both hypophosphatemic groups after restoring plasma P_i_ concentrations. Furthermore, *V*_ATP_ was directly related to cellular and mitochondrial P_i_ uptake in L6 and RC13 rodent myocytes and isolated muscle mitochondria. Similar findings were observed in a patient with chronic hypophosphatemia as a result of a mutation in *SLC34A3* who had a 50% reduction in both serum P_i_ content and muscle *V*_ATP_. After oral P_i_ repletion and normalization of serum P_i_ levels, muscle *V*_ATP_ completely normalized in the patient. Taken together, these data support the hypothesis that decreased muscle ATP synthesis, in part, may be caused by low blood P_i_ concentrations, which may explain some aspects of muscle weakness observed in patients with hypophosphatemia.—Pesta, D. H., Tsirigotis, D. N., Befroy, D. E., Caballero, D., Jurczak, M. J., Rahimi, Y., Cline, G. W., Dufour, S., Birkenfeld, A. L., Rothman, D. L., Carpenter, T. O., Insogna, K., Petersen, K. F., Bergwitz, C., Shulman, G. I. Hypophosphatemia promotes lower rates of muscle ATP synthesis.

Inorganic phosphate (P_i_) is essential for membrane structure, energy storage, and signal transduction in all cells (1), and homeostatic mechanisms exist to maintain extracellular P_i_ concentration in the physiologic range, which varies with age and from species to species (2). Acute hypophosphatemia can cause rhabdomyolysis and respiratory and heart failure, which often complicates care of patients in the intensive care setting (3, 4). Chronic hypophosphatemia leads to rickets or osteomalacia, which is often complicated by impaired muscle function and early fatigue, a feature that significantly affects quality of life (5). A possible connection between blood P_i_, muscle energy metabolism, and vitamin D status has been suggested by studies that have demonstrated a delayed phosphocreatine recovery time in patients who are hypophosphatemic secondary to vitamin D deficiency, although it is unclear from these studies whether these changes could also be attributed to effects of vitamin D deficiency (6).

The contribution of P_i_ to regulation of respiratory control and mitochondrial substrate oxidation was recognized some decades ago (7–9), but ADP has been considered the most important regulator of mitochondrial respiration, with P_i_ acting only as a coregulator (10), which binds to specific sites on ATP synthase (11). Tanaka *et al* (12) showed that P_i_ is rate limiting for mitochondrial function in the perfused liver and Beard *et al* (13) suggested that P_i_ controls mitochondrial respiration, in particular, during low-to-moderate oxygen consumption.

Type III transporters SLC20A1/Pit1 and SLC20A2/Pit2 serve as ubiquitous P_i_ transporters in all tissues (14). Genetic ablation of Pit1 in all tissues is lethal at embryonic d 12.5 and causes abnormal liver development and defects in erythroid and B-lymphocyte differentiation (15); however, Pit1-knockout mice have normal development of skeletal muscle, likely as a result of compensation by Pit2, as shown by [^32^P] uptake experiments using mutant Pit1-null mouse embryonic fibroblasts (15). Mice and humans who lack Pit2 are viable and their skeletal muscle seems to be normal, but they develop brain calcifications later in life (16, 17). As there is no obvious muscle phenotype in the transporter knockouts, it remains unclear whether extracellular P_i_ modifies muscle function *via* homeostatic mechanisms, that is, synthesis of 1,25(OH)_2_-D (18), or *via* changes in intracellular P_i_.

Muscular energy requirements are met through hydrolysis of ATP, which is mainly synthesized by mitochondrial oxidative phosphorylation. Rates of muscle ATP synthetic flux (*V*_ATP_) can be noninvasively assessed by using saturation transfer [^31^P]-magnetic resonance spectroscopy (ST-[^31^P]MRS) (19–23). By using this method, we have previously demonstrated that muscle *V*_ATP_ is reduced in healthy elderly subjects and in insulin-resistant offspring of parents with type 2 diabetes (20, 21). In addition, it has been shown that muscle *V*_ATP_ doubles during insulin stimulation in insulin-sensitive individuals and that this response is blunted in the insulin-resistant offspring (22). However, how alterations in P_i_ affect muscle function and muscle ATP synthetic flux is poorly understood. To further explore the association between P_i_, muscle function, and ATP synthetic flux, we studied the relation between plasma P_i_ and muscle *V*_ATP_ measured by ST-[^31^P]MRS in diet- and genetically induced hypophosphatemic mice and in a patient with chronic hypophosphatemia as a result of hereditary hypophosphatemic rickets who had a 50% reduction in serum P_i_ content.

## MATERIALS AND METHODS

### Reagents

Chemicals were obtained from commercial sources and used as received, unless noted otherwise.

### Animals

All rodent protocols were approved by the Yale University School of Medicine Animal Care and Use Committee. Mice were housed under controlled temperature (22 ± 2°C) and lighting (12 h of light, 0700–1900 h; 12 h of dark, 1900–0700 h), with free access to water and food (0.7% P_i_, 1.0% Ca, TD 2018S; Harlan Teklad, Madison, WI, USA).

### *NaPi2a^−/−^* mouse model

Mice homozygous for a mutation in the gene of sodium-dependent P_i_ transporter solute carrier family 34, member 1 (*NaPi2a*) were obtained from The Jackson Laboratory (Bar Harbor, ME, USA) and bred with C57BL/6J mice. Resulting heterozygotes (HETs) were bred HET by HET, and *NaPi2a^−/−^* and wild-type (WT) littermate controls were used for experiments.

**Figure 1.**
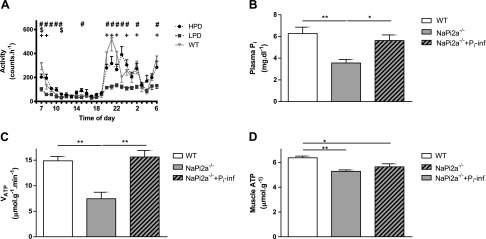
*NaPi2a^−/−^* mice were studied at age 5–6 mo after receiving LPD (0.02% P_i_, 0.6% Ca) or HPD (1.2% P_i_, 0.6% Ca) for 10 wk. *A*) CLAMS was used to determine activity over a period of 72 h for LPD and HPD, which was compared with WT littermate controls on regular chow (RC). Activity was reduced in mice that received LPD and was mostly normalized after administering HPD. *B*, *C*) Mice with genetically induced hypophosphatemia (*NaPi2a^−/−^*) were hypophosphatemic compared with WT littermate controls (*B*) and showed reduced *V*_ATP_ determined by ST-[^31^P]MRS (*C*). *D*) Actual muscle ATP concentration measured by LC-MS was decreased in *NaPi2a^−/−^* mice compared with WT littermate controls. Plasma P_i_ levels and VATP normalized to euphosphatemic levels after infusion of a bolus of 25 μmol P_i_ (*B*, *C*), whereas muscle ATP concentration was not changed after infusion (*D*). All data are means ± sem; *n* = 5–8 in each group. ^+^*P* < 0.05, *NaPi2a^−/−^* on LPD *vs*. WT on RC; ^$^*P* < 0.05, *NaPi2a^−/−^* on HPD *vs*. WT on RC; ^#^*P* < 0.01, NaPi-2a^−/−^ on LPD *vs*. HPD; *P < 0.05; **P < 0.01 by double-sided Student’s *t* test.

For experiments shown in **Fig. 1** and **Table 1**, *NaPi2a^−/−^* mice were evaluated at age 2–3 mo, placed on a low-P_i_ diet (LPD; 0.02% P_i_, 0.6% Ca, egg white–based, TD 140659; Harlan Teklad) or a high-phosphate diet (HPD; 1.2% P_i_, 0.6% Ca, egg white–based, TD 85349; Harlan Teklad), and evaluation was repeated at 2 and 10 wk. Plasma P_i_ and lactate were measured at baseline, 2 wk, and 10 wk on LPD and HPD. Comprehensive animal metabolic monitoring system (CLAMS; see below) was used to determine activity and respiratory exchange ratio over a period of 72 h of mice that were fed *ad libitum* with LPD and HPD.

**TABLE 1.** Biometric and metabolic data assessed by CLAMS of NaPi2a^−/−^ mice on LPD and HPD for 10 wk and of WT mice on RC and LPD for 2 wk

Parameter	*NaPi2a^−/−^* diet for 10 wk				WT diet for 2 wk		
	WT on RC	LPD	HPD	*P* (LPD *vs*. HPD)	RC	LPD	*P* (RC *vs*. LPD)
Body weight (g)	27.4 ± 0.5	25.7 ± 0.9	28.8 ± 1.3		28.1 ± 0.9	26.1 ± 0.3	
Muscle mass (g)	20.5 ± 0.5	17.4 ± 0.5	19.1 ± 0.6	0.05	21.4 ± 0.3	20.3 ± 0.2	0.03
Fat mass (g)	1.5 ± 0.2	2.9 ± 0.2	4.3 ± 0.7		3.0 ± 0.3	2.1 ± 0.3	
Muscle mass (%)	74.9 ± 0.7	67.8 ± 0.5	67.0 ± 1.2		76.4 ± 2.0	77.6 ± 0.9	
Fat mass (%)	5.5 ± 0.8	11.0 ± 0.7	14.0 ± 1.7		10.6 ± 0.8	8.1 ± 1.0	
*V*_O2_ (ml/kg/h)	2972.4 ± 38.0	3439.4 ± 93.6	3350.0 ± 136.8		3300.9 ± 27.7	3422.2 ± 25.0	<0.01
*V*_CO2_ (ml/kg/h)	2719.7 ± 38.0	3145.5 ± 79.3	3112.7 ± 149.0		3166.9 ± 24.8	3408.5 ± 27.7	<0.01
RER	0.91 ± 0.01	0.91 ± 0.01	0.92 ± 0.01		0.96 ± 0.01	0.99 ± 0.01	<0.01
Energy expenditure (Kcal/kg/h)	14.6 ± 0.2	17.0 ± 0.4	16.2 ± 0.5		16.5 ± 0.1	17.2 ± 0.1	<0.01
Energy intake (Kcal/kg/h)	17.5 ± 0.5	23.2 ± 2.4	23.2 ± 1.2		26.7 ± 1.5	36.6 ± 2.5	<0.01
Activity index (counts/min)	158.5 ± 19.4	78.1 ± 8.9	109.7 ± 7.8	0.01	52.5 ± 2.4	44.1 ± 1.5	<0.05

All data are means ± sem; *n* = 8–10 in each group. *P* values are indicated when significant by double-sided Student’s *t* test. RC, regular chow (0.7% P_i_, 1.0% Ca). See Fig. 1 for composition of LPD and HPD.

### LPD-induced hypophosphatemic mouse model

**Figure 2.**
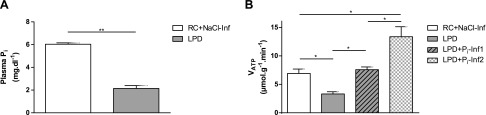
*A*) WT mice 12–18 wk old maintained on LPD for 2 wk become hypophosphatemic. Mice received a bolus of 30 μmol P_i_ (P_i_-Inf1), 60 μmol (P_i_-Inf2), and saline for control group (NaCl-Inf) while in the magnet. *B*) *V*_ATP_ determined by ST-[^31^P]MRS was reduced in LPD mice, was increased in these animals after P_i_-Inf1, and further increased after P_i_-Inf2 compared with NaCl-infused mice. See Fig. 1 and Table 1 for composition of LPD and regular chow (RC). All data are means ± sem; *n* = 5–8 in each diet group. *P < 0.05, **P < 0.01 by double-sided Student’s *t* test.

Male C57BL/6J mice (The Jackson Laboratory) between 2 and 3 mo of age were individually housed and maintained on either standard mouse chow diet with 0.6% P_i_ (RC; TD2018; Harlan Teklad) or LPD with 0.02% P_i_ and 0.6% calcium (TD96817; Harlan Teklad). Mice were matched for age, weight, and food withdrawal time within each experiment, and, if possible, between experiments (**Fig. 2** and Table 1).

### Mouse ST-[^31^P]MRS

Muscle mitochondrial ATP synthetic flux in mice was assessed by ST-[^31^P]MRS by using a 9.4 T superconducting magnet system (horizontal/10-cm diameter bore magnet, 400.55 MHz for proton; Magnex Scientific, Palo Alto, CA, USA) interfaced to a Varian console as described earlier (19, 24, 25). Proof-of-principle studies have been performed in rat hind limb (24, 25) and humans (26) to validate the method. In brief, the animal was anesthetized by using 1.5% isoflurane to prevent movement during the experiment, the left hind limb was shaved, and the mouse was positioned prone in a stereotactic frame to ensure consistent, reproducible positioning for each study using a homemade holder that exposed the muscle to concentric surface coils [an outer ^1^H coil (25 mm) and an inner [^31^P] coil (15 mm)]. This stable experimental setup eliminates movement of the region of interest and guarantees acquisition of high signal/noise [^31^P] spectra. Body temperature was maintained at 37°C by using a heated cradle incorporated into the MR probe, and optimal signals were acquired by using localizers to ensure accurate positioning of the animal in the magnet. To monitor cardiopulmonary status of the animal throughout the experiment and control depth of anesthesia to maintain stable physiologic function, a multiparameter pulse oximeter was used (MouseOx Plus; Starr Life Sciences, Holliston, MA, USA). For shimming, a FastMap sequence over a 2.8- × 2.8- × 2.8-mm volume of the leg was applied. Coil geometries were optimized to match the size of the mouse and to ensure close proximity of the muscle to the coil for a high signal-to-noise ratio while avoiding occlusion of blood flow to the mouse extremity. By using Nuts software (Acorn NMR, Livermore, CA, USA) for spectra processing, we are able to calculate *V*_ATP_. With the help of fully relaxed [^31^P] spectra, we estimated muscle P_i_ content. Parameters were calibrated against ATP and P_i_ measurements that were obtained from muscle tissue after dissection of animals (Fig. 1*D*). ST-[^31^P]MRS measurements were started 1 h after P_i_ infusion. Each animal was measured before and after P_i_ infusion. See Supplemental Data for more information.

### P_i_ infusion of *NaPi2a^−/−^* and LPD mice

**Figure 3.**
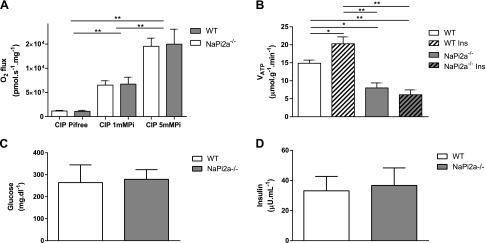
*A*) *In vitro* measurement of oxygen flux in isolated skeletal muscle mitochondria of WT and *NaPi2a^−/−^* mice increased in a dose-dependent manner upon addition of 1 and 5 mM Pi to medium; however, mitochondrial function *per se* is not impaired in *NaPi2a^−/−^* mice compared with WT. *B*) Insulin increased *V*_ATP_ in WT but not in *NaPi2a^−/−^* mice when mice were infused with 5 mU/kg/min insulin, followed by 3 mU/kg/min of insulin and 12.5 mg/kg/min of 20% glucose at a rate of 2.1 μl/min on the basis of body weight of the animal to maintain euglycemia and hyperinsulinemia during ST-[^31^P]MRS (hyperinsulinemic-euglycemic clamp). *C*, *D*) Plasma glucose (*C*) and insulin (*D*) values measured after the experiment were not different between the 2 groups. CIP, oxidative phosphorylation capacity of complex I–related substrates. All data are means ± sem; *n* = 5–8 in each group. *P < 0.05; **P < 0.01 by double-sided Student’s *t* test.

*NaPi2a^−/−^* mice at age 2–3 mo were studied after 2 wk on LPD (0.02% P_i_, 0.6% Ca, TD96817; **Fig. 3**). Before ST-[^31^P]MRS experiment, mice were infused with a bolus of 25 μmol NaH_2_PO_4_ (pH 7.4) on the basis of a 72 ml/kg blood volume for a mouse to restore normophosphatemia (∼5.6 mg/dl).

WT mice were divided into 3 groups that each received a phosphate infusion on the basis of the protocol used for *NaPi2a^−/−^* mice while inside the MRS instrument. The LPD group received a bolus of 30 μmol NaH_2_PO_4_ and the HPD group received 60 μmol NaH_2_PO_4_ at pH 7.4. Control group received 60 μmol of iso-osmotic NaCl infusion. Blood samples were obtained at end of study by cardiac puncture for plasma [P_i_] measurements.

### Hyperinsulinemic-euglycemic clamp conditions

*V*_ATP_ was assessed during hyperinsulinemic-euglycemic conditions as shown in Fig. 3*B*. At the beginning of this study, we infused a bolus of 5 mU/kg/min of insulin, followed by 3 mU/kg/min of insulin (Novo Nordisk, Bagsværd, Denmark) at a rate of 2.1 μl/min and 12.5 mg/kg/min of 20% dextrose to maintain euglycemia during hyperinsulinemia. Blood samples were taken retro-orbitally after the clamp. At study completion, mice were anesthetized and tissues were harvested within 3 min with liquid N_2_–cooled aluminum tongs and stored at –80°C for subsequent analysis.

### Case report of the patient with hypophosphatemic rickets with hypercalciuria

The study was approved by the Yale University Human Investigation Committee and written informed consent was obtained from the participant after explanation of the purpose, nature and potential complications of the study. We measured *V*_ATP_ and serum P_i_ before, after 5 mo, and again after a total of 8 mo oral P_i_ treatment to normalize plasma phosphorous concentrations in a 28-yr-old male individual with hereditary hypophosphatemic rickets with hypercalciuria (HHRH), a genetic form of hypophosphatemia as a result of loss-of-function of the proximal tubular sodium-P_i_ cotransporter NaPi2c, as previously reported by us (27). The patient initially presented at age 14 yr with gradual onset of bilateral knee and ankle pain. At age 13 yr, he had passed his first calcium oxalate kidney stone. There were bilateral genua valga on initial examination and he walked with a limp. He was found to have hypophosphatemia, elevated urinary calcium to creatinine ratio, and an increased 1,25(OH)_2_D level, whereas the concentration of 25-hydroxyvitamin D and midmolecule parathyroid hormone were within normal limits [see Bergwitz *et al*. (27) for more details]. Because he was poorly compliant with P_i_ supplements, his serum phosphorus level remained in the 1.5–2.0 mg/dl range, his adult height was 159 cm well below midparental height, and he has had multiple episodes of symptomatic nephrolithiasis. He had not been taking P_i_ supplements for at least 12 mo before initial evaluation. The patient presented before diagnosis and treatment with moderately severe muscle weakness. He tired rapidly after attempting to play sports with his peers and responded well with resolution of these complaints after 2–4 wk of supplemental phosphate therapy, after which time he could maintain normal activity. Spectroscopy studies were performed after being treated for several years, but in the midst of a limited period of nonadherence with phosphate medication, and, unfortunately, no formal muscle strength quantification was available at the time of study. Subjectively, he noticed improvement with therapy after periods of missing medication. Hypophosphatemia was restored by oral P_i_ supplementation with 500 mg KPhos MF 3 times/d, with a total daily dose of 1500 mg for a period of 8 mo. Serum P_i_ and *V*_ATP_ were assessed in 5 healthy age-matched males who served as control group.

### CLAMS

CLAMS (Columbus Instruments, Columbus, OH, USA) was used to evaluate oxygen consumption (*V*_O2_), carbon dioxide production (*V*_CO2_), and respiratory exchange ratio (RER) as well as energy expenditure, food consumption, and activity for body weight and fat-matched animals as previously described (28). Fat and lean body masses were assessed by ^1^H-MRS (Bruker BioSpin, Billerica, MA, USA). Energy expenditure and RER were calculated from gas exchange data (energy expenditure = [3.815 + 1.232 × RER] × *V*_O2_). RER is the ratio of *V*_CO2_ to *V*_O2_, which changes depending on the substrate that the animal is using. When carbohydrates are the only substrate being oxidized, RER will approximate 1.0, and when only fatty acids are metabolized, 0.7.

### Human ST-[^31^P]MRS

ST-[^31^P]MRS was performed as previously described (26) in a 4.0 Tesla whole-body MR scanner interfaced to a Bruker Avance console (Bruker Biospin). In brief, patients were positioned supine in the magnet with an MR probe that consisted of a 9-cm diameter [^31^P] surface coil and a concentric 13-cm diameter ^1^H surface coil placed over the right calf muscle. B0 field of the magnet was optimized by shimming using the FastMap procedure, and the voxel of interest was positioned inside the soleus muscle after imaging of the calf. Analysis of [^31^P] data was performed by using MatLab software (MathWorks, Natick, MA, USA); Free induction decays were zero-filled and apodized using a 5 Hz Lorentzian function before Fourier transformation. Spectra were manually phased and baseline corrected by fitting to a fifth-order polynomial. Peak areas of [^31^P] metabolites were determined by integration. Concentrations of [^31^P] metabolites were determined from fully relaxed spectra assuming a constant 5.5 mmol/kg muscle ATP concentration.

### Calculation of muscle mitochondrial ATP synthetic flux

*T*_1_ of P_i_ under conditions of γ-ATP saturation (*T*_1_′ - spin lattice relaxation time) was measured by using an adiabatic 7-point inversion recovery pulse sequence in the presence of continuous γ-ATP saturation to calculate *k*′ (Eq. 1). Unidirectional ATP synthetic flux was calculated as *k*′ × [P_i_] (Eq. 2). P_i_ concentration was assessed from a fully relaxed baseline MRS spectrum obtained at a TR of 35 s (comparing peak integrals from P_i_ and γ-ATP). Saturation-transfer parameters were: 2 ms adiabatic half passage excitation pulse (centered between P_i_ and γ-ATP), 15 s soft saturation pulse, sweep width = 10 kHz, 4096 points, effective repetition time = 15 s.
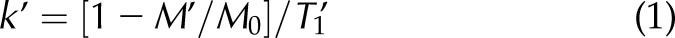

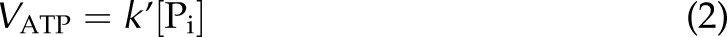
where *M*′ is P_i_ signal during γ-ATP saturation; and *M*_0_ is P_i_ signal without γ-ATP saturation.

### *In vitro* ATP synthesis from isolated mitochondria

For isolation of mitochondria from the hind limb of *NaPi2a^−/−^* and WT mice (Fig. 3*A*), excised muscle was placed in 1 ml mitochondrial isolation buffer MiB06 (100 mM sucrose, 100 mM KCl, 50 mM Tris-HCl, 1 mM KH_2_PO_4_, 0.2 mM EFTA, fatty acid–free albumin from bovine serum) and cut into pieces. After washing 3–4 times with 1 ml MiB06, 1 ml of protease solution (2 mg/10 ml MiB06) was added and incubated for 2 min. Sample was then homogenized, transferred to a centrifuge tube, and centrifuged at 800 *g* for 10 min at 4°C. Supernatant was transferred to a new centrifuge tube after filtering through a cheesecloth and centrifuged at 10,000 *g* for 10 min at 4°C. Supernatant was discarded and mitochondrial pellet was resuspended in MiB06. After determining mitochondrial protein concentration by Bradford assay, intact mitochondria corresponding to 10 mg of mitochondrial protein were added to the chamber of an a oxygraph 2K (Oroboros Instruments, Innsbruck, Austria) that contained P_i_-free mitochondrial respiration buffer MiR05 (0.5 mM EGTA, 3 mM MgCl_2_0.6H_2_O, 60 mM potassium-lactobionate, 20 mM taurine, 20 mM HEPES, 110 mM sucrose, and 1 g/L bovine serum albumin at pH 7.1) and oxygen flux (pmol/s/ml) was measured at 37°C. Standardized instrumental calibrations were performed to correct for back-diffusion of oxygen into the chamber from various components, leak from exterior, oxygen consumption by chemical medium, and sensor oxygen consumption. Glutamate (10 mM), malate (2 mM) and ADP (1 mM) was added to the P_i_-free medium to favor complex I respiration. Oxygen flux by complex I in dependence of different concentrations of P_i_ was determined by using DatLab (Oroboros).

### Cell culture

L6 cells (passage number 5–20) were grown at 37°C under 5% CO_2_ in 150 cm^2^ flasks in 15-ml α-minimal essential medium with 1% penicillin/streptomycin and 2% fetal bovine serum (FBS) after growth to confluency (myocytes). RC13 cells (passage number 5–10) were grown at 37°C under 5% CO_2_ in 150 cm^2^ flasks in 15 ml DMEM that was supplemented with 10% FBS and 1% penicillin/streptomycin. For experiments, cells were grown to confluency in 24-well plates. Medium was purchased from Thermo Fisher Scientific Life Sciences (Waltham, MA, USA). Medium was changed every 3–4 d and cells were passaged (1:10) every 5–7 d after removal from the plate with 1 ml trypsin/EDTA. For experiments, cells were grown to confluency in 12- or 24-well plates in medium that contained 2% FBS and were differentiated into myotubes for 10–20 d.

### Western blots

Cell lysates were analyzed by PAGE and immunoblotted for phosphorylated Akt (p-Akt) and total Akt as previously described (28) by using an antibody that detects p-Ser473 of Akt (Cell Signaling Technology, Danvers, MA, USA), which is the amino acid phosphorylated by PI3K and activates Akt.

### Phosphate and glucose uptake assays

Cells were starved in serum-free α-minimal essential medium for 14–16 h before the experiment. Cells were then treated with indicated concentrations of inhibitors for 20 min: wortmannin (500 nM) and LY294002 (50 µM; Calbiochem, San Diego, CA, USA), KP372-1 (100 nM; Echelon Biosciences, Salt Lake City, UT, USA), and Akt-I-1/2 (100 nM; Sigma-Aldrich, St. Louis, MO, USA). ETOH and DMSO were used as vehicle controls. Inhibitors were delivered to each well in 1-µl volumes. After treatment with an inhibitor or vehicle control, cells were treated with 100 nM insulin (Novo Nordisk) or 5 µl H_2_O for 30 min. Wells were then rinsed with 500 µl buffer A 3 times. Each well received 500 µl of transport buffer B and was incubated for exactly 10 min. Reactions were quenched by washing each well with 500 µl of ice-cold buffer A for P_i_ transport and buffer A + 5 mM d-glucose for glucose transport. Cells were lysed by treatment with 400 µl of 1 M NaOH and neutralized with 400 µl of 1 M HCl. In both assays, radioactivity was quantified by liquid scintillation counting, normalized to protein content determined by BCA protein assay (Thermo Fisher Scientific Life Sciences), and reported as moles of P_i_ or glucose transported per minute per mg protein.

### Data analysis

All results are presented as means ± sem. Multiple comparisons were analyzed by ANOVA—followed, when appropriate, by Turkey *post hoc* test—by using Prism 6 for Windows software (GraphPad Software, La Jolla, CA, USA). Single comparisons were performed by 2-tailed unpaired Student’s *t* test. For statistical analysis of human patient data, means of replicate measurements were compared with reference range. A value of *P* ≤ 0.05 was considered statistically significant.

## RESULTS

### Reduced spontaneous exercise capacity in *NaPi2a^−/−^* mice

Excessive renal loss of P_i_ in *NaPi2a*^−/−^ mice was reflected by a 50% reduction in plasma P_i_ (3.6 ± 0.1 compared with 6.3 ± 0.2 mg/dl in WT littermates; *P <* 0.001) and decreased further to 2.1 ± 0.3 mg/dl (*P <* 0.001 *vs*. baseline) after 2–10 wk on LPD (see Materials and Methods). Plasma P_i_ levels normalized after 3–10 wk on HPD (see Materials and Methods; 5.8 ± 1.5 mg/dl; *P <* 0.001 *vs*. baseline). Spontaneous physical activity, as assessed over 72 h using CLAMS, was reduced by ∼30% in *NaPi2a^−/−^* after 10 wk on LPD compared with HPD (LPD: 78 ± 9 counts/h *vs*. HPD: 110 ± 8 counts/h; *P =* 0.014; Table 1 and Fig. 1*A*). Similarly, after 2 wk on LPD, WT mice had reduced spontaneous activity (LPD WT: 44.1 ± 1.5 counts/h *vs*. WT: 52.5 ± 2.4 counts/h; *P =* 0.006) and increased RER (WT LPD: 0.99 ± 0.01 *vs*. WT: 0.96 ± 0.01; *P =* 0.001; Table 1). On LPD, WT mice lost 5% muscle mass (*P =* 0.03 *vs*. WT) and *NaPi2a*^−/−^ mice lost 15% (*P =* 0.05 *vs*. *NaPi2a*^−/−^ on HPD; Table 1). Furthermore, both caloric intake and energy expenditure were increased in WT mice on LPD. Food intake and energy expenditure were similar between groups (Table 1). All mice appeared well as determined by normal grooming behavior and body weight.

### *NaPi2a^−/−^* mice are hypophosphatemic and have reduced muscle *V*_ATP_ *in vivo*

On the basis of our prior ST-[^31^P]MRS studies in humans that showed that insulin stimulation of muscle mitochondrial *V*_ATP_ correlated with intracellular P_i_ concentrations (22), we next tested the hypothesis that hypophosphatemia decreases muscle P_i_ uptake and *V*_ATP_ in *NaPi2a*^−/−^ mice. Higher magnetic field strengths now allow for *in vivo* ST-[^31^P]MRS in the small volumes of hind limb muscle of mice (∼0.8 cm^3^) (19, 29), and by further optimizing acquisition of [^31^P]MRS of the muscle, insulin-clamp studies could be performed over 4–6 h.

**Figure 4.**
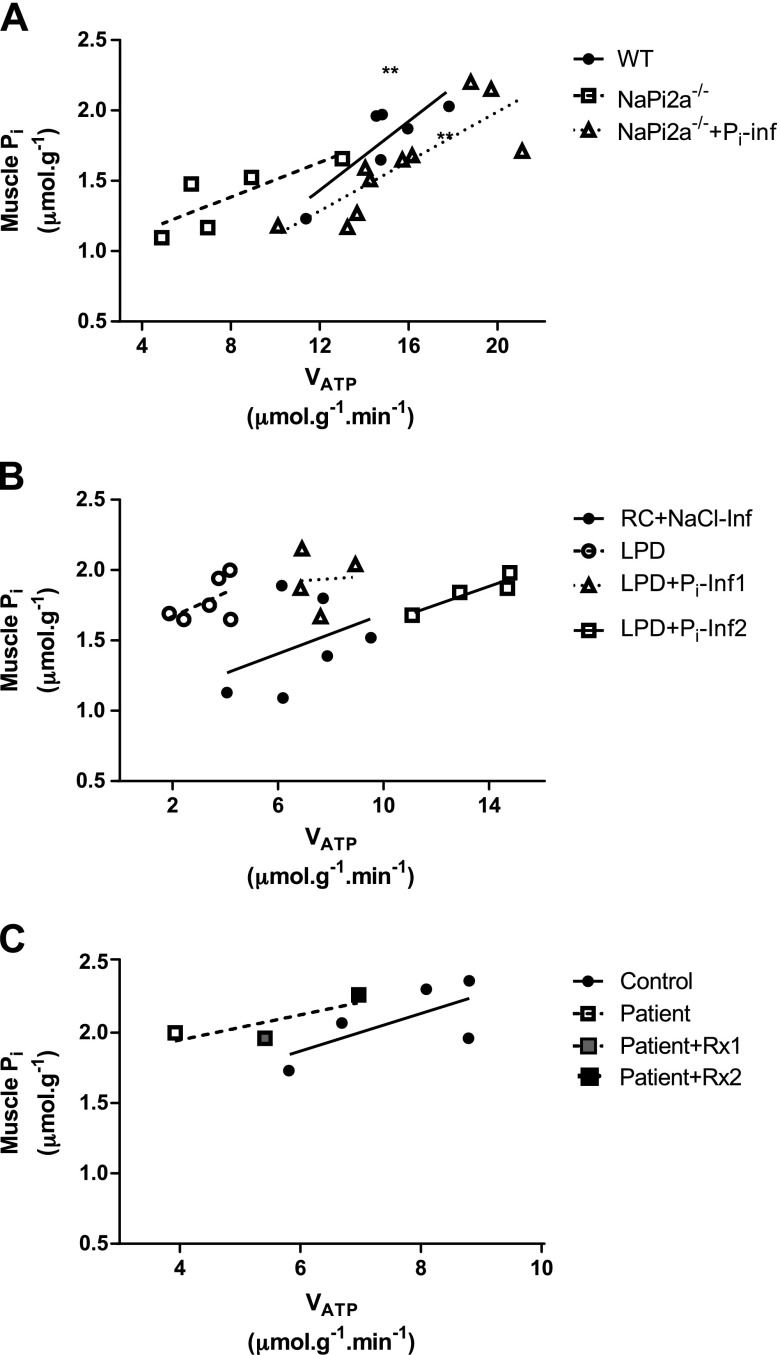
Muscle P_i_ concentration and *V*_ATP_ are positively correlated. Shown is a regression analysis of muscle P_i_ and *V*_ATP_ obtained in WT mice, *NaPi2a^−/−^* mice, and *NaPi2a^−/−^* mice after continuous P_i_ infusion (*A*), in the LPD model before and after P_i_ infusion (*B*), and in the patient with hypophosphatemic rickets (*C*). P_i_ infusion (P_i_-Inf) in panels *A*, *B* and oral phosphate supplementation after 5 (Rx1) and 8 (Rx2) mo improves *V*_ATP_ in all 3 models. Shown are individual animals with regression lines for each treatment group. Pooled regression analysis of all treatment groups was significant with **P < 0.01 in each panel.

*NaPi2a*^−/−^ mice were hypophosphatemic with a 43% reduction in plasma P_i_ (3.6 ± 0.1 *vs*. 6.3 ± 0.2 mg/dl in WT; *P* < 0.01; Fig. 1*B*). Muscle mitochondrial *V*_ATP_ was 50% lower in *NaPi2a*^−/−^ mice than in WT (7.4 ± 1.3 *vs*. 14.9 ± 0.9 µmol/g/min; *P* = 0.001; Fig. 1*C*). Correspondingly, muscle ATP concentration measured by liquid chromatography–tandem mass spectrometry was reduced in *NaPi2a*^−/−^ mice compared with their WT littermate controls (6.4 ± 0.1 *vs*. 5.3 ± 0.1 µmol/g; *P* = 0.007; Fig. 1*D*). There was a positive correlation between *V*_ATP_ and muscle P_i_ concentrations in WT mice (*R*^2^ = 0.72; *P* = 0.03) and a similar trend for *NaPi2a^−/−^* mice (*R*^2^ = 0.65; *P* = 0.10; **Fig. 4*A***). On the basis of observed rate constant *k*′ reductions in *NaPi2a^−/−^* mice, the diminished *V*_ATP_ seemed to be a result of reduced ATP synthase activity rather than its level of expression in the mitochondrial membrane (Supplemental Fig. S3*F*).

### Phosphate infusion restores plasma and muscle P_i_ and normalizes *V*_ATP_ in *NaPi2a^−/−^* mice

Bolus infusion of *NaPi2a^−/−^* mice with 25 µmol NaH_2_PO_4_ (pH 7.4) resulted in normalization of plasma P_i_ from 3.6 ± 0.1 to 5.6 ± 0.2 mg/dl (*P* < 0.001) and led to complete normalization of muscle mitochondrial *V*_ATP_ (from 7.4 ± 1.3 to 15.6 ± 1.3 µmol/g/min; *P* = 0.001; Fig. 1*B*, *C*). Muscle ATP concentrations were not changed after infusion (Fig. 1*D*). Muscle P_i_ concentrations correlated with *V*_ATP_ in *NaPi2a^−/−^* mice after NaH_2_Po_4_ infusion (*R*^2^ = 0.68; *P* = 0.004; Fig. 4*A*). Pooled analyses of all 3 groups (WT and *NaPi2a^−/−^* pre- and post-NaH_2_PO_4_) show that muscle *V*_ATP_ is directly correlated with muscle P_i_ concentrations (*R*^2^ = 0.55; *P* < 0.001; Fig. 4*A*).

### Muscle mitochondrial *V*_ATP_ is low in mice on LPD

When WT C57Bl/6 mice were fed LPD for 2 wk to induce hypophosphatemia, similar results were observed, with a 65% reduction in plasma P_i_ concentrations from 6.0 ± 0.1 to 2.1 ± 0.3 mg/dl on LPD (*P* < 0.001). *V*_ATP_ in these mice was reduced by 52% compared with the regular chow-fed group (3.3 ± 0.4 *vs*. control: 6.9 ± 0.8 µmol/g/min; *P* = 0.002; Fig. 2).

### Phosphate infusion restores plasma P_i_ and normalizes *V*_ATP_ in hypophosphatemic mice

To reverse hypophosphatemia and examine the effects on muscle mitochondrial *V*_ATP_, mice on LPD were given bolus infusions of 30 and 60 µmol NaH_2_P0_4_ (pH 7.4) at 2 µl/min. Basal *V*_ATP_ in hypophosphatemic mice on LPD was 6.9 ± 0.8 µmol/g/min and increased in a dose-dependent manner to 7.6 ± 0.5 µmol/g/min (*P* < 0.001 *vs*. preinfusion) after low-dose P_i_ infusion and 1.9-fold after high-dose P_i_ infusion to 13.4 ± 0.9 µmol/g/min (*P <* 0.01 *vs*. preinfusion; Fig. 2*B*). Linear regression analysis of pooled data for all groups again showed a positive correlation between muscle P_i_ and *V*_ATP_ (*R*^2^ = 0.55; *P* < 0.001; Fig. 4*B*). On the basis of the observed changes of the rate constant *k*′, this increase in *V*_ATP_ likely reflects increased physiologic ATP synthase activity rather than higher expression of ATP synthase in the inner mitochondrial membrane, which would be reflected by an increase in *V*_max_ (Supplemental Fig. S3*C*).

### Oxygen flux depends on external P_i_ concentration in isolated mouse mitochondria

To directly test the impact of P_i_ concentration on oxidative phosphorylation capacity *in vitro*, we measured the rate of oxygen flux in isolated quadriceps mitochondria from WT and *NaPi2a^−/−^* mice in a P_i_-free medium in the presence of glutamate, malate, and ADP. Oxygen flux increased upon addition of 1 mM P_i_ and further increased upon addition of 5 mM P_i_ to medium (Fig. 3*A*). Increase in oxygen flux was similar between the 2 groups, which suggests that loss of *NaPi2a* and hypophosphatemia *per se* does not impair muscle mitochondrial function.

### Insulin stimulation of *V*_ATP_ is impaired in *NaPi2a^−/−^* mice

To examine the potential role of plasma P_i_ levels on insulin stimulation of muscle mitochondrial *V*_ATP_ a hyperinsulinemic-euglycemic clamp was performed in WT and *NaPi2a^−/−^* mice during ST-[^31^P]MRS to directly measure basal and insulin-stimulated muscle *V*_ATP_, which increased by 23% in the WT group (basal: 14.9 ± 0.9 *vs*. insulin-stimulated: 20.3 ± 1.9 µmol/g/min; *P* = 0.03). In contrast, insulin stimulation had no effect on *V*_ATP_ in *NaPi2a^−/−^* mice (Fig. 3*B*). Plasma glucose and insulin values were not different between the 2 groups (Fig. 3*C*, *D*). Saturation transfer effect (*M*′/*M*_0_), effective MR relaxation time of P_i_ (*T*_1_′) and the rate constant *k*′ were not changed in response to insulin stimulation but were different between WT and *NaPi2a^−/−^* mice (Supplemental Fig. S3*G*–*I*).

### Insulin increases P_i_ uptake into L6 and RC13 myocytes that are dependent on PI3K-Akt signaling

We have previously observed a strong relationship between insulin stimulation of intramyocellular P_i_ concentrations and muscle *V*_ATP_ in healthy, lean, insulin-sensitive control subjects and a severe blunting of this effect in otherwise healthy, young, lean, insulin-resistant offspring of parents with type 2 diabetes (T2D) (22). To further examine this relationship between muscle P_i_ concentrations and *V*_ATP_, [^32^P]_i_ uptake was stimulated with 100 nM insulin in L6 human myocytes in the presence of sodium (0.012 ± 0.001 *vs*. 0.025 ± 0.001 pmol PO_4_/mg/min with insulin; *P* < 0.001; Supplemental Fig. S1*A*). Stimulation of P_i_ uptake was dose dependent and inhibited by wortmannin, a potent and specific PI3K inhibitor. Similar results were obtained by using the PI3K inhibitor LY294002 (50 μmol/min) and the PDK1 inhibitor KP372-1 (100 nM; Supplemental Fig. S1*B*, *C*), whereas Akt-I-1/2, an inhibitor of Akt, had no effect on blocking P_i_ uptake (Supplemental Fig. S1*D*) when used at a dose of 120 nM, which is sufficient to inhibit glucose uptake by GLUT4 in these cells (Supplemental Fig. S2*D*). These data suggest that insulin-stimulated glucose transport involves activation of PI3K, PDK1, and Akt, whereas insulin-stimulated P_i_ transport only involves PI3K and PDK1. Similar results were obtained in human RC13 myocyte cells (Supplemental Fig. S1*E*, *F*).

### Muscle *V*_ATP_ in a patient with a mutation in the NAPI2C gene

**Figure 5.**
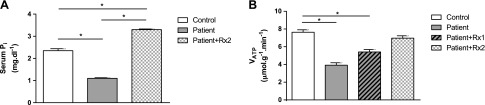
An individual with hypophosphatemic rickets has reduced *V*_ATP_. *A*) Serum P_i_ was measured before and again after restoration of hypophosphatemia after a total of 8 mo (Rx2) oral phosphate supplementation with 500 mg KPhos MF 3 times/d, which comprised a total daily dose of 1500 mg. *B*) *V*_ATP_ was measured before, after 5 mo (Rx1), and again after a total of 8 mo (Rx2) treatment. The patient had reduced *V*_ATP_ measured by ST-[^31^P]MRS before treatment compared with 5 healthy controls, but *V*_ATP_ was restored after 8 mo treatment. All data are means ± sem; *n* = 5 in the control group. *P < 0.01 by double-sided Student’s *t* test.

Hereditary HHRH is a genetic form of hypophosphatemia that is caused by loss of the proximal tubular sodium P_i_ cotransporter NaPi2c (30). We measured serum P_i_ concentrations and *V*_ATP_ by using ST-[^31^P]MRS after an overnight fast in 5 healthy volunteers and in 1 patient with HHRH before and after 8 mo of oral treatment with 500 mg KPhos MF administered 3 times/d. Baseline serum P_i_ concentrations in the patient with HHRH were 1.1 mg/dl (normal range, 2.5–4.5 mg/dl) and increased to 3.3 mg/dl after P_i_ supplementation. At baseline, muscle *V*_ATP_ was reduced by 50% in the patient with HHRH (3.9 *vs*. 7.6 ± 0.3 µmol/g/min for healthy controls) and normalized with serum P_i_ after treatment to 5.4 and, finally, 7.0 µmol/g/min (Fig. 5). Muscle P_i_ concentrations were 2.0 μmol/g before and increased to 2.3 μmol/g after chronic oral P_i_ supplementation.

## DISCUSSION

By using ST-[^31^P]MRS technology, we found that mice with both genetically and diet-induced hypophosphatemia have reduced spontaneous activity and that this is accompanied by an ∼50% decrease in plasma P_i_ concentrations and muscle *V*_ATP_. We also found that muscle *V*_ATP_ is directly related to cellular and mitochondrial P_i_ uptake in L6 and RC13 rodent myocytes and isolated muscle mitochondria, and demonstrate that both serum P_i_ concentrations and muscle *V*_ATP_ were reduced by 50% compared with healthy control individuals in a patient with hereditary HHRH and that muscle *V*_ATP_ completely normalized after oral P_i_ supplementation. Therefore, decreased muscle ATP synthesis, in part, may explain muscle weakness associated with hypophosphatemia and may serve as a noninvasive marker for hypophosphatemic myopathy.

Hypophosphatemia causes severe muscle weakness in many organisms, including humans (31, 32), dogs (33), and rodents (34). As muscle function in hypophosphatemic individuals rapidly returns to normal when physiologic P_i_ levels are restored (35), muscle weakness is likely directly related to blood P_i_ concentration, although it is possible that other changes in bone and mineral metabolism caused by hypophosphatemia are involved (36). Effects of P_i_ on muscle function as a substrate for biologic processes, such as energy metabolism and signal transduction, are not well understood. It is also not known which metabolic sensing pathways regulate uptake of P_i_ into cells and subcellular compartments, such as mitochondria.

We used murine knockout of *NaPi2a*, which is hypophosphatemic as a result of renal loss of P_i_ (37), as a model of hypophosphatemic myopathy. These mice have reduced spontaneous activity and impaired ATP synthesis, which leads to a metabolic shift toward glycolytic ATP generation (38).

To directly determine rates of muscle ATP synthetic rate in *NaPi2a^−/−^* mice, we adapted ST-[^31^P]MRS to mice (24, 25, 39). By using ST-[^31^P]MRS, we found that muscle *V*_ATP_ is markedly reduced in *NaPi2a^−/−^* mice. We reproduced these findings in a patient with hypophosphatemic myopathy and in WT mice in which hypophosphatemia was induced by LPD. Furthermore, *V*_ATP_ in all our models studied was rapidly restored after P_i_ supplementation consistent with the fact that VATP is measuring ATP synthetic flux but not steady state levels, and therefore a more sensitive to measure Pi effects on muscle metabolism. These findings suggest that *V*_ATP_ is likely directly related to blood P_i_ concentration.

P_i_ enters muscle cells *via* type III Na/P_i_ cotransporters (P_i_T1 and P_i_T2) (40). We therefore next evaluated whether blood P_i_ influences *V*_ATP._ We observed that muscle P_i_ correlates with *V*_ATP_ (Fig. 4) before and after P_i_ repletion. This suggests that muscle P_i_, which is the sum of interstitial and intracellular, but probably mostly intramyocellular, is an important determinant of mitochondrial phosphorylation activity.

To further test whether *V*_ATP_ is related to intramyocellular P_i_ in *NaPi2a^−/−^* mice, we examined isolated muscle mitochondria from these mice. Oxygen flux, a measure of *V*_ATP_, is stimulated when P_i_ is added to the assay buffer; however, no difference was observed between mitochondria isolated from WT and *NaPi2a^−/−^* mice, which suggests that intramyocellular P_i_ regulates *V*_ATP_ and that intramyocellular P_i_ as a result of hypophosphatemia is likely the cause of reduced *V*_ATP_ in these mice.

In support of our earlier observations that insulin sensitivity correlates with muscle P_i_ and *V*_ATP_ in insulin-sensitive and insulin-resistant individuals (22), we demonstrated that insulin stimulates P_i_ uptake into cultured myocytes—evident in 2 cell lines—in a PI3K- and PDK1-dependent fashion. Furthermore, by using ST-[^31^P]MRS, we demonstrated that insulin stimulates muscle P_i_ and *V*_ATP_ in WT mice by 21%, whereas this stimulation was absent in hypophosphatemic *NaPi2a^−/−^* mice. This finding also supports the notion that muscle P_i_ measured by ST-[^31^P]MRS predominantly reflects intramyocellular Pi, extends our observation in isolated mitochondria, and further supports the hypothesis that muscle P_i_ uptake is important for *V*_ATP_.

Finally, we showed in a patient with chronic hypophosphatemia as a result of a loss-of-function mutation in the NaPi2c renal phosphate transporter gene (*SLC34A3*) that the 50% reduction in plasma Pi was accompanied by a 50% reduction in muscle *V*_ATP_ and also that normalization of plasma P_i_ levels with oral phosphate repletion resulted in complete normalization of muscle *V*_ATP_.

Taken together, our data strongly suggest that cellular P_i_ uptake is an important determinant of *V*_ATP_ and hypophosphatemic myopathy in mice and men. Future studies in mice in which P_i_ transporters are selectively deleted in muscle will further clarify the role of P_i_ uptake for mitochondrial and muscle function.

Our findings may have broader implications for 2 common clinical conditions: refeeding syndrome and myopathy commonly observed in T2D. Refeeding syndrome is a potentially fatal complication that occurs in patients with starvation, malnutrition or alcohol abuse (41). Although well recognized, this condition remains underdiagnosed and the exact mechanisms are not fully understood. Our data suggest that P_i_ transport into myocytes is stimulated by insulin, a hormone markedly induced during refeeding, which may provide a pathophysiologic explanation to the hypophosphatemia observed.

Muscle strength and function are also impaired in T2D (42, 43). Although peripheral diabetic neuropathy, in part, explains this weakness, exact reasons remain unclear. We recently showed that these individuals have decreased muscle P_i_ content and decreased insulin-stimulated rates of ATP synthesis (22), which suggests that insulin requires normophosphatemia to maintain normal *V*_ATP_. Furthermore, in RC13 rhabdomyosarcoma cells and L6 myocytes, insulin regulates P_i_ uptake upstream of Akt, which involves activation of PI3K and PDK1. Similar observations were made by Wang *et al* (44), who reported increased rat vascular smooth muscle cell calcification after selective inhibition of the PI3K pathway while intact insulin signaling attenuated vascular smooth muscle cell calcification induced by high P_i_ conditions by the ability of insulin to reduce serum P_i_ concentrations and promote redistribution of P_i_ from the circulation into the cell. On the basis of these considerations, decreased muscle ATP synthetic flux may serve as a noninvasive marker to monitor therapy of patients with refeeding syndrome and T2D.

Our findings suggest that decreased muscle ATP synthetic flux, in part, may explain muscle weakness in hypophosphatemia, and *V*_ATP_ may serve as a noninvasive marker for hypophosphatemic myopathy. Our findings further establish insulin as a potential regulator of muscle P_i_ transport and of *V*_ATP_. These findings might help to better understand the pathophysiology of refeeding hypophosphatemia and of the myopathy commonly observed in individuals with T2D.

## Abbreviations

CLAMS: comprehensive animal metabolic monitoring system
FBS: fetal bovine serum
HET: heterozygote
HHRH: hypophosphatemic rickets with hypercalciuria
HPD: high-phosphate diet
LPD: low-phosphate diet
*NaPi2a*: sodium-dependent P_i_ transporter solute carrier family 34, member 1
P_i_: inorganic phosphate
RER: respiratory exchange ratio
ST-[^31^P]MRS: saturation transfer [^31^P]-magnetic resonance spectroscopy
T2D: type 2 diabetes
*V*_ATP_: ATP synthetic flux rate
WT: wild-type
